# Filling Capacity Evaluation of Self-Compacting Concrete in Rock-Filled Concrete

**DOI:** 10.3390/ma13010108

**Published:** 2019-12-25

**Authors:** Wenju Liu, Jianwen Pan

**Affiliations:** State Key Laboratory of Hydroscience and Engineering, Tsinghua University, Beijing 100084, China; wj-liu13@outlook.com

**Keywords:** rock-filled concrete, self-compacting concrete, filling capacity, grain shape

## Abstract

The good filling performance of self-compacting concrete (SCC) to pre-placed assembly of rocks is essential for quality of rock-filled concrete (RFC). In this study, a theoretical model is proposed to evaluate the filling capacity of SCC in porous media that is simplified to approximate the assembly of rocks. Numerical simulation of SCC flow in the porous media is carried out based on the computational fluid dynamics. The effects of yield stress of SCC and size and shape of grains in the porous media on the filling capacity of SCC are considered. The inclination of the free surface of the distribution of SCC at flow stoppage is defined to evaluate the filling capacity of SCC in the porous media. According to the theoretical model, the inclination is directly proportional to the yield stress of the SCC and the blocking effect of grains, while inversely proportional to the grain size. The numerical simulation provides consistent results with the theoretical model. The results suggest the use of rounded large rocks and SCC with low yield stress to ensure good quality of RFC.

## 1. Introduction

Rock-filled concrete (RFC) [1] is a new type of mass concrete and it is formed by filling self-compacting concrete (SCC) into the voids between a pre-placed assembly of rock grains. SCC has high flowability, and gravity alone can quickly fill the pores of the assembly of large rocks more than 300 mm in grain size. RFC has obvious advantages in terms of the absence of vibration or compaction during the construction process compared with conventional concrete or roller-compacted concrete, and thus it leads to a shorter construction duration, lower intensive of work and less cost, as well as environmentally friendly materials [2]. From the material point of view, RFC combines the advantages of masonry and concrete, and it decreases cement consumption, lowers temperature rise of hydration heat, and reduces shrinkage of concrete. RFC has become a promising material for large scale structures. Over one hundred RFC dams have been built in China [3]. The quality of the RFC is dominated by the filling content of SCC in the pores of the assembly of rocks, and a dense filling is expected for good RFC of high strength and durability. Therefore, the filling capacity of SCC into the pre-placed assembly of rocks, which is recognized as a complicated porous medium, is crucial and needs to be evaluated.

The filling performance of SCC into porous media mainly depends on two factors: (1) the rheological properties (viscosity and yield-stress), and (2) the characteristics of grain accumulation including the grain scale and shape. The workability properties can be evaluated by using different testing methods, including the slump flow test and V-funnel test. However, an experiment alone is not capable for full evaluation of the workability properties of SCC, for instance, the slump flow test is used to assess the horizontal free flow of SCC and allows to estimate the yield-stress [4,5], while the V-funnel test is applied to evaluation of flowability and segregation and allows to estimate the viscosity [6].

Passing ability between closely spaced obstacles is one of the most important performance characteristics of SCC since it determines the final filling capacity, which influences the strength and durability of hardened SCC [7]. The influence parameters of passing ability involve the rheological properties of SCC, aggregate size and shape, volume of aggregates, and clearance between reinforced bars [8]. Various techniques were developed for evaluating the passing ability of SCC through closely spaced obstacles. L-Box and J-Ring tests are widely adopted to assess the passing ability of SCC through reinforcing bars in concrete structures [9]. However, it may not be suitable for evaluating the passing ability of SCC flowing in the assembly of rock grains since the closely spaced obstacles of the tests consist of only one layer of reinforcing bars, which are not competent to represent the complex voids of the assembly of rocks. Therefore, new methods are important for the filling performance evaluation of SCC considering the complex assembly of rock grains. Xie et al. [3] conducted an experiment to preliminarily investigate the filling capacity of SCC in the assembly of rocks considering the effect of aggregate size and yield stress of SCC. Wang et al. [10] proposed a Grating-Box test, in which the grain packs are composed of cylindrical obstacles, to assess passing ability and filling performance of self-compacting mortar (SCM). But the effect of grain shape on the filling performance of self-compacting mortar on the porous media is neglected in the test method.

In addition to testing methods, numerical procedures have also been proposed to investigate the filling performance of SCC. The computational fluid dynamic (CFD) is widely used for flow property analysis of fresh SCC due to its efficiency and flexibility [11,12,13]. The research suggested the CFD method is suitable to predict the flow behavior of fresh SCC using the Bingham or Herschel–Bulkley fluids models. Many studies have been conducted to investigate the passing ability of SCC using numerical methods. Hosseinpoor et al. [14] employed the CFD to simulate the blocking resistance of SCC. They considered the effect of reinforcing bar spacing and coarse aggregate content. Cui et al. [15] proposed the discrete element method (DEM) to study the blocking phenomenon when SCC flows through reinforcing bars. The results showed that aggregate size is the most important factor affecting the passing ability of SCC under similar coarse aggregate volume fraction. Chen et al. [16] presented a coupled LBM-DEM model to simulate SCC flowing in the porous media with multi-channels. They found that jamming of SCC occurs when the spacing between the channel is less than 2 times the aggregate size. However, the volume of coarse aggregates in SCC is usually conditioned to the passing ability [8]. Therefore, if the minimum spacing of the voids in the porous media is greater than 2 times the coarse aggregate size and there is suitable coarse aggregate volume in SCC, blockage of SCC due to coarse aggregates can be neglected and thus SCC can be seen as a homogeneous material flowing in the porous media. On this basis, Vasilic et al. [17] treated SCC as a homogeneous fluid and the reinforcement as a porous medium, which could then simplify the pre-processing and reduce the computational time. The authors suggested an equivalent permeability of the steel bars network for evaluation of the flow resistance of SCC. Shin et al. [18] followed this approach and proposed a generalized model to predict the filling ability of SCC in steel-plate concrete panels. In the above mentioned studies, the obstacles are reinforcing bars and the evaluation of passing ability of SCC ignores the effect of obstacle shape. The filling performance of SCC flowing in porous media, especially an assembly of rocks, is lacking an evaluation method.

The blockage of coarse aggregates will not occur during the placement of SCC in the pre-placed assembly of rocks since the spacing between the rocks is generally greater than ~2–3 times the maximum size of the aggregates in SCC. SCC can be approximated as a homogeneous fluid flowing through the complicated porous medium composed of rocks, and thus its filling capacity depends on the rheological properties of SCC and the pore structures of the medium.

The purpose of this study is to evaluate the filling capacity of SCC in an assembly of rocks in the casting process of RFC. The assembly of rocks is simplified using porous boxes composed of grains. The SCC used in this study is regarded as a homogeneous fluid and simulated with the Herschel–Bulkley fluid model. Grains of different sizes and shapes to characterize the assembly of rocks are considered. A theoretical model is first derived to evaluate the filling capacity of SCC flowing in the porous media. The CFD method is then applied to simulate the flow of SCC in the porous boxes, and the evaluation of filling capacity is verified with the theoretical model. The effects of the rheological properties of SCC and the size and shape of grains on the filling capacity of SCC in the porous media are discussed.

## 2. Theoretical Model for Filling Capacity of SCC

### 2.1. Porous Boxes

**Figure 1:**
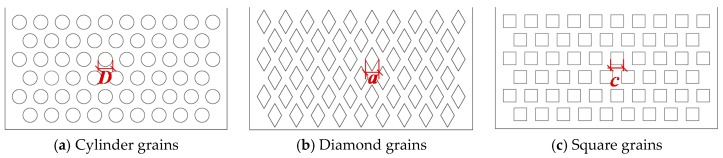
Porous boxes with different shaped grains. (**a**) Cylinder grains; (**b**) Diamond grains; (**c**) Square grains.

The porous box is proposed to simulate the assembly of rocks in the RFC. The porous box is composed of grain packs as shown in Figure 1. Three different shapes of grains, including cylinder, square column and diamond column, are considered. The grains of each shape have three different sizes with a cross-sectional area of 113.1 mm^2^, 201.1 mm^2^, and 314.2 mm^2^, respectively. The parameters of the nine porous boxes are presented in detail in Table 1. The inner thickness of the nine porous boxes is the same and is *b* = 100.00 mm. The porosity of all the porous boxes is approximately 0.66 and thus its effect on the flow of SCC is not discussed in this study.

**Figure 2:**
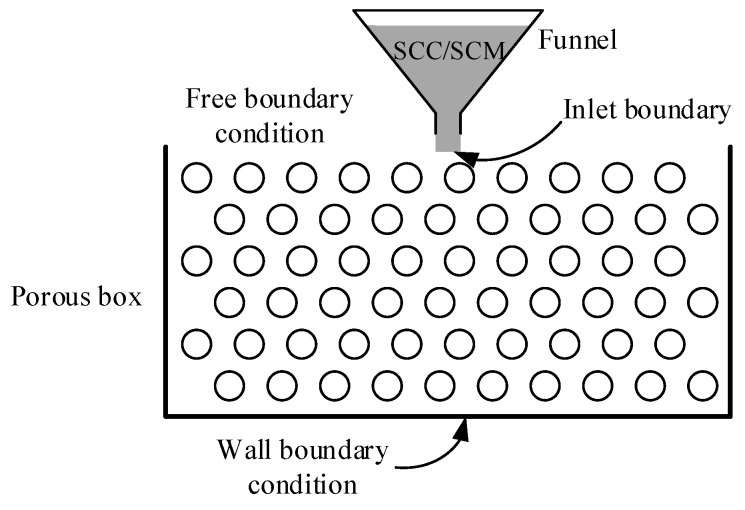
Illustration of pouring of self-compacting mortar (SCM) into the porous box and boundary condition of the numerical simulation.

SCC is approximated as a homogeneous fluid and SCM is used to simulate the flow behavior of SCC in the porous box. The SCM is poured on the top of the porous box, as shown in Figure 2. The SCM has a constant volume that is determined according to the funnel. The V-funnel volume in this study is 1.134 L. The SCM flows through the voids in the porous box, and fills the pores under the effect of gravity. Its final distribution in the porous box at flow stoppage is defined to represent the filling capacity of SCM in the porous media.

### 2.2. Numerical Model for SCC

SCC can be seen as a homogeneous fluid when it flows in the assembly of large rocks during the casting process of RFC since the voids between the rocks are large enough compared with the aggregates of SCC such that blockage of coarse aggregates is neglected. Therefore, the continuum approach is suitable to model the SCC flowing. The Bingham or Herschel–Bulkley (H–B) fluid has been widely used to describe flows of SCC and SCM [19,20,21]. In this study, the H–B model which has been implemented in OpenFoam is adopted. The shear stress τ and shear rate $\dot{\gamma }$ has a nonlinear relationship.

(1) $$\tau =\tau_{0}+k{\dot{\gamma }}^{m}$$

where $\tau_{0}$ is the yield stress, *k* is the consistency index, and *m* is the power law index. In this study, *m* is assigned as 1. The apparent viscosity η is written:

(2) $$\eta (\dot{\gamma })=\frac{\tau_{0}}{\dot{\gamma }}+k{\dot{\gamma }}^{m-1}$$

The H–B model is implemented into the computational fluid dynamic software OpenFoam [22]. The numerical simulation of flows of SCC and SCM is performed using the software OpenFoam in this study. The interFoam solver, which is based on the Volume of Fluid (VOF) method, is used. The initial and boundary conditions for the numerical model are shown in Figure 2. The wall of the porous box is set as a wall boundary condition. The top of the porous box is a free boundary. The inlet boundary for the SCC/SCM inflow is set in the center of the top and has a size of 30 mm × 100 mm. The inlet velocity is 0.03 m/s and the inflow time is 12.6 s. After the SCC/SCM flow entering the box, the inlet boundary is modified to a free boundary. The mesh size of the simulation domain is 2 mm. A desktop computer, which has an i7-6700hq and 8 GB memory, is used for the analysis. The calculation time for one case ranges from 2 h to 5 h. The results of the simulation are processed with Paraview [23].

The Grating-Box test proposed by Wang et al. [10] is employed to verify the numerical model. In the experimental tests, the grating-box was composed of cylinder. Three boxes were designed with different grain sizes, which are identical with box C_I, C_II and C_III (Table 1). The Grating-Box test was done using SCM to simulate the behavior of SCC. The proportion of the SCM mixtures can be found in Wang et al. [10].

Numerical simulations of different SCM flowing in the porous boxes are carried out. SCMs with different spreading radii, which were measured using the slump flow tests [10], are considered for the corresponding cases. The numerical simulation cases and the rheological parameters of the SCM are shown in Table 2. The yield stress of SCM can be determined [4] with the empirical Equation (3):

(3) $$\tau_{0}=\frac{225\rho gV^{2}}{128\pi^{2}R^{5}}$$

where *ρ* is the density of the fresh SCM and is taken 2355 kg/m^3^ in this study, *g* is gravity acceleration, *V* is the volume of the cone used in the slump flow test, and *R* is the spreading radius of the SCM.

After the yield stress $\tau_{0}$ is calculated, the consistency index *k* is determined by adjusting the numerical simulation of mini-cone test to make the flow stoppage time be consistent with the laboratory experiment.

**Figure 3:**
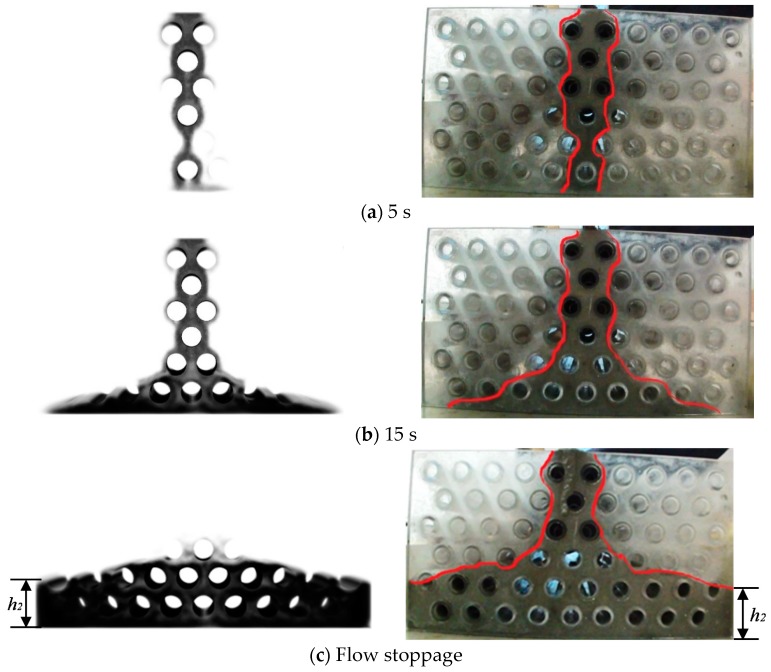
The flow process of SCM in the porous box (left: simulation; right: test [10]). (**a**) 5 s; (**b**) 15 s; (**c**) Flow stoppage.

Figure 3 illustrates the typical flow process of the SCM in the porous box. It can be seen that the numerical simulation result is similar to the experimental result though a small amount of SCM is observed adhering to the wall of the porous box at the end of the flow. The SCM is poured on the top of the porous box, and flows through the voids under gravity effect and reaches the bottom of the porous box at 5 s. The SCM accumulates at the bottom and spreads forwards both the left and right sides of the porous box. As the flow stops, the SCM fills the pores at the bottom, forming a pile with a slope on the free surface. The pile of SCM, so called final distribution in this study, is to represent the filling capacity of SCM in the porous media.

**Figure 4:**
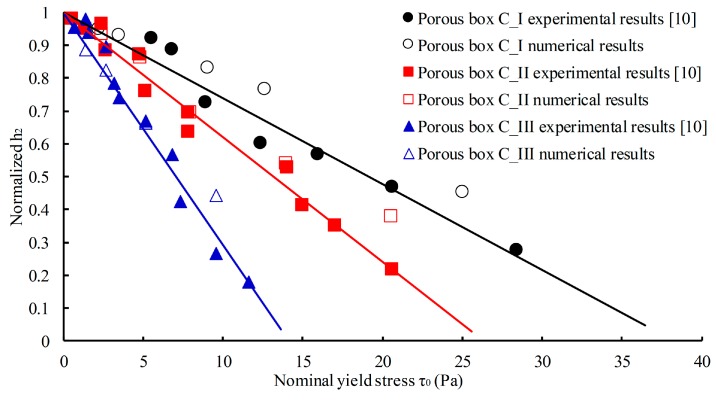
Normalized *h*_2_ of SCM distribution versus yield stress of SCM.

The SCM distribution height *h*_2_, which is defined as the average height of the left and right sides of the final distribution of SCM in the porous box, is introduced to evaluate the filling capacity of SCM. The SCM distribution height *h*_2_ was normalized by dividing with the *h*_max_ represents the calculated height of SCM when the SCM has a horizontal surface in the porous box, i.e., *h*_max_ = *V*/*bL*_0_*φ*, where *V* denotes the volume of SCM, *b* and *L*_0_ are the thickness and length of boxes, and *φ* is the porosity of the box. Figure 4 shows the relationship between the normalized *h*_2_ of SCM distribution and the yield stress of the SCM. It demonstrates that the normalized *h*_2_ and the yield stress of SCM has a linear relationship for each porous box. The numerical results are in good agreement with the experimental results. Therefore, the numerical model of SCM can perform simulation of SCM flowing in porous media with sufficient accuracy.

### 2.3. Derivation of the Theoretical Model

The SCC distribution in the porous box at flow stoppage, representing the filling capacity of SCC in the porous media, is investigated. To obtain the relationship between the SCC distribution and the pore structure of the porous box, the following assumptions were made:

(a) SCC is regarded as a homogeneous fluid.
(b) When the flow stops, the SCC distribution in the porous box is symmetrical and its free surface in the cross-section is regarded as a straight line.
(c) When the flow is about to stop, the main flow direction is horizontal and the vertical velocity is negligible.

**Figure 5:**
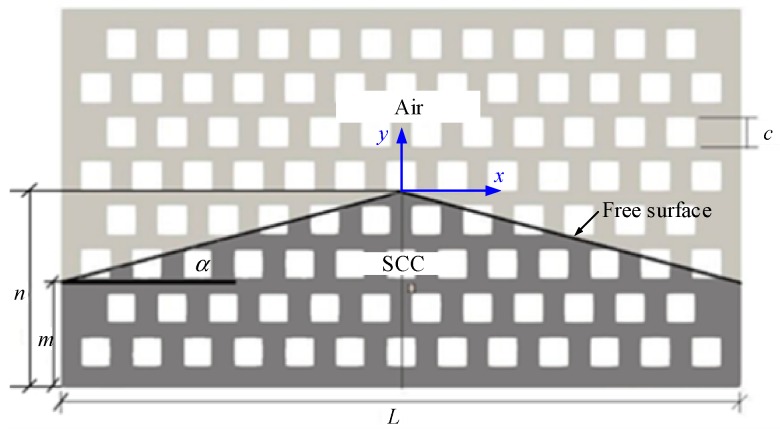
Self-compacting concrete (SCC) distribution in the porous box at the flow stoppage.

Taking the porous box with square columns for example, when the flow of the SCC stops, the final distribution of SCC is formed, as illustrated in Figure 5. The inclination of the free surface of the final distribution is defined for evaluation of the filling capacity of SCC.

(4) $$I=\tan\alpha =-\frac{dy}{dx}$$

where *α* is the angle between the free surface of the final distribution and the horizontal plane.

**Figure 6:**
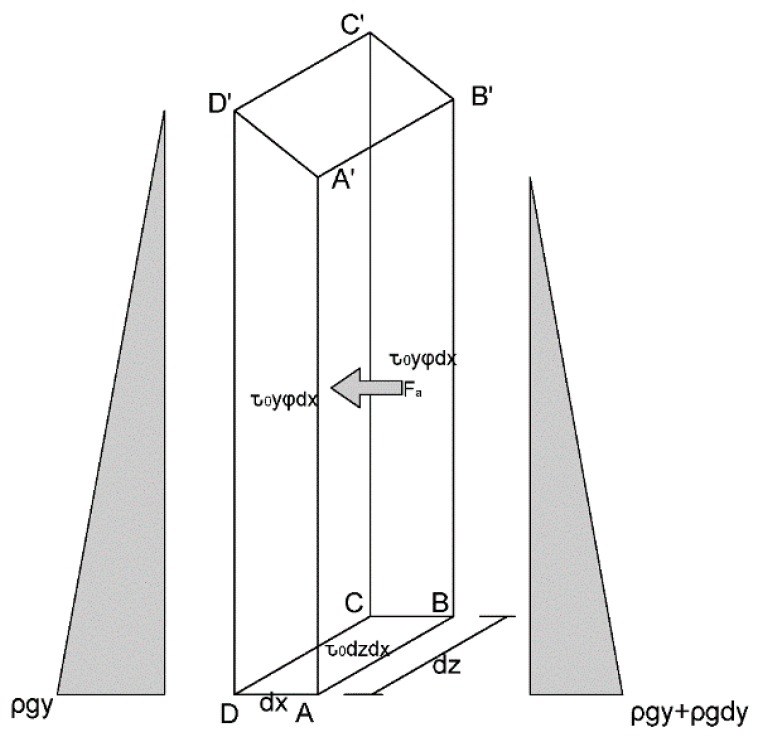
The forces of the mortar isolator at flow stoppage.

An isolator with a thickness of d*z* and a width of d*x* is truncated from the final distribution of the SCC. The forces of the SCC isolator are shown in Figure 6. In accordance with static equilibrium, Equation (5) is derived:

(5) $$\int_{0}^{y}\rho gydy-\int_{0}^{y+\mathrm{dy}}\rho gydy=\tau_{0}y\varphi dx+\tau_{0}y\varphi dx+\tau_{0}dzdx+F_{a}$$

where *ρ* is the density of SCC, *τ*_0_ is the yield stress of SCC, *g* is the gravity acceleration, *φ* is the porosity of the porous box, and *F_a_* is the static drag force generated by the grains.

Equation (5) can be simplified and is written:

(6) $$-\rho gydy=\tau_{0}dx(2y\varphi +dz)+F_{a}$$

Brookes and Whitmore [24] suggested that when the Bingham fluid flows through the barrier, the static drag force can be calculated as follows:

(7) $$F_{a}=KA\tau_{0}$$

where *A* represents the projected area in the direction orthogonal to the flow direction of the fluid, and *K* is a constant parameter.

The drag force in the isolator is assumed to distribute evenly in the SCC, and it can be calculated:

(8) $$\;F_{a}=KN_{S}A_{S}\tau_{0}$$

where $N_{S}=(1-\varphi )(y+y+dy)dxdz/(2c^{2}dz)$ is the number of the square columns immersed in the SCC isolator, *A_S_* = *c*d*z* is the horizontal projected area, in which *c* is the size of the square column. Therefore, the drag force *F_a_* is obtained:

(9) $$F_{a}=\frac{K(1-\varphi )\tau_{0}}{c}ydzdx$$

Substituting Equation (9) into Equation (6), the force balance equation is rewritten as follows:

(10) $$-\rho gydy=\tau_{0}dx(2y\varphi +dz)+\frac{K(1-\varphi )\tau_{0}}{c}ydzdx$$

Therefore, the inclination of the free surface of the final distribution is obtained

(11) $$I_{S}=\frac{\tau_{0}}{\rho g}(2\varphi +\frac{dz}{y})+\frac{\tau_{0}}{\rho g}\frac{K(1-\varphi )dz}{c}$$

in which, the two terms on the right-hand side are, respectively, the boundary resistance and statistic drag force. The term (2*φ* + d*z*/*y*) has the magnitude of 1.0 due to the porosity *φ* < 1.0, whereas the term *K*(1 − *φ*)d*z*/*c* is much greater since the statistic drag force caused by obstacles is obviously greater than the boundary drag force. The boundary resistance is thus neglected. Therefore, the inclination of the free surface of the final distribution in the porous box with square columns is written as:

(12) $$I_{S}=\frac{\tau_{0}}{\rho g}\frac{K(1-\varphi )dz}{c}$$

Similarly, the inclination of the SCC distribution in the porous boxes with cylinder and diamond columns are given by:

(13) $$I_{C}=\frac{\tau_{0}}{\rho g}\frac{4K(1-\varphi )dz}{\pi D}$$

(14) $$I_{D}=\frac{\tau_{0}}{\rho g}\frac{2K(1-\varphi )dz}{a}$$

For the quasi-3D boxes, the thickness d*z* = 1, a general formula of the inclination of the free surface of the SCC distribution is derived:

(15) $$I=\frac{\tau_{0}}{\rho g}\frac{MK(1-\varphi )}{s}$$

where *M* is the grain shape-related parameter, and *s* is the grain size. *MK* describes the blocking effect of the grains on the SCC flowing in the porous media. It is found that the inclination of the free surface of the final distribution is directly proportional to the yield stress of SCC and is inversely proportional to the size of the grains.

## 3. Results and Discussions

### 3.1. Effect of Yield Stress on Filling Capacity of SCC

The SCC filling in the porous boxes with different pore structures forms a similar geometry of the final distribution at the bottom. The inclination of the free surface of the SCC distribution at flow stoppage is used as an indicator to evaluate the SCC filling capacity in the porous media. Generally, a decrease in inclination indicates the SCC with higher flowability and better filling performance in the porous media, but the proper range for inclination values needs further investigation combining with in-field engineering practice.

Table 3 lists the predicted inclination of the free surface of the SCC distribution in the nine different porous boxes using the numerical simulation. Six cases for each box are considered, in which the SCC has different yield stress, i.e., 16.72 Pa, 18.84 Pa, 21.20 Pa, 23.55 Pa, 28.26 Pa, and 35.33 Pa.

**Figure 7:**
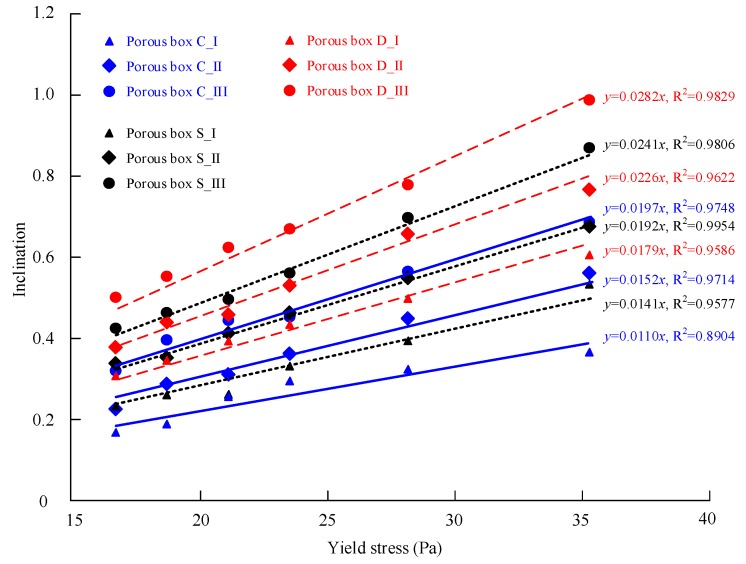
The inclination–yield stress relationship for the porous boxes.

Figure 7 shows the relationship between the inclination and the yield stress for the porous boxes with cylinder, square and diamond grains. It is obvious that a horizontal free surface of the distribution would be obtained when using a fluid with zero yield stress (such as water) to fill the porous boxes. In other words, the inclination is zero at zero yield stress. Therefore, a straight line with a y-axis intercept of zero is used to fit the numerical simulation data points. The correlations coefficient R^2^ ranges from 0.89 to 0.98 for all the porous boxes. The results show that the inclination of the SCC distribution in the porous media is directly proportional to the yield stress of the SCC. Besides, for the same shape of grains in the porous box, the inclination increases as the grain size decreases. This finding is identical to the theoretical formula of the inclination.

According to the results, the SCC has a better filling capacity as its yield stress is smaller. For the same SCC and the porous media with similar porosity, the SCC exhibits better filling capacity in the porous media with greater size of grains. Therefore, the SCC with low yield stress and large rocks are expected to ensure good quality of RFC.

### 3.2. Effect of Grain Scale and Shape on Filling Capacity of SCC

According to the proposed theoretical formula of inclination of the SCC distribution in the porous box, the inclination *I* depends on the shape of the grains (*M*), the size of the grains (*s*) and the constant parameter (*K*). The value of *K* can be determined from the numerical simulation results and are listed in Table 4. The values of *K* are close for the porous boxes with the same shape of grains, implying that the parameter *K* represents the characteristics of grain accumulation and is not affected by the pore scale in the porous media. The average values of the blocking effect of grains *M*$\bar{K}$ for the porous boxes with cylinder, square and diamond grains are 152.91, 170.40 and 219.24, respectively. It implies that the diamond grains having greater *M*$\bar{K}$ value results in worse filling capacity of SCC in the porous media.

**Figure 8:**
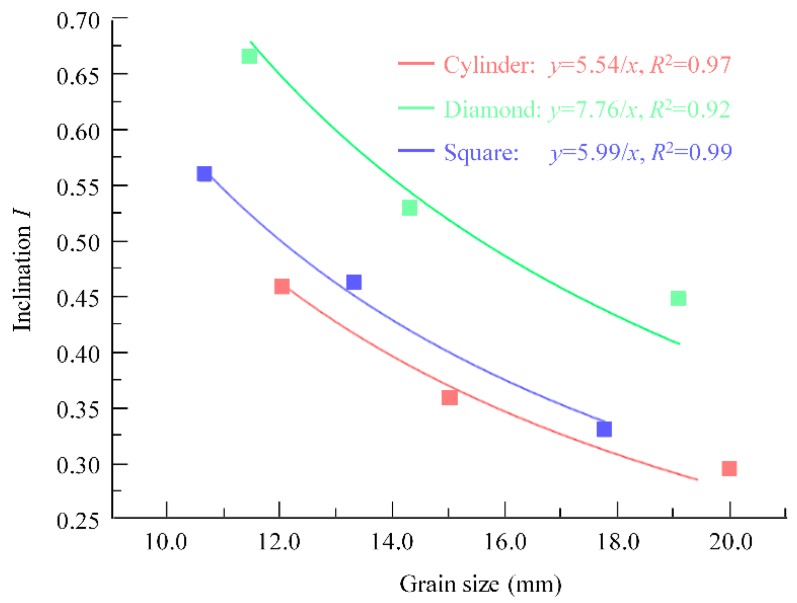
The relationship of inclination and aggregate size (*τ*_0_ = 23.55 Pa).

Figure 8 shows the relationship between the inclination and the grain size obtained from the numerical simulation. Only the case of yield stress $\tau_{0}$ = 23.55 Pa is given, and the other cases with different yield stresses are similar and are not shown. It can be seen that the inclination is inversely proportional to the size of the grains. This phenomenon can be observed in the different porous boxes with different shapes of grains. The numerical result is in good agreement with the theoretical model in terms of the inversely proportional relationship between inclination and grain size. Therefore, for the same SCC, grains with bigger sizes in the porous media lead to a better filling capacity of the SCC.

It can also be found in Figure 8 that the inclination for cylinder grains is lowest compared with that for square or diamond grains, indicating that SCC exhibits better filling performance in porous media composed of grains with smooth shapes. This observation is consistent with the *M*$\bar{K}$ value of the theoretical model.

## 4. Conclusions

This study investigates the filling capacity of self-compacting concrete (SCC) in a pre-placed assembly of large rocks in the casting process of rock-filled concrete (RFC). A theoretical model is proposed to evaluate the filling capacity of SCC in the porous media. The effects of yield stress of SCC and shape and size of grains in the porous media are considered. Numerical simulations based on the computational fluid dynamics are carried out. The results from numerical simulation together with the theoretical model are applied to analyze the filling performance of SCC in porous media. The main conclusions are summarized below:

(1) The inclination *I* of the free surface of SCC distribution at flow stoppage is defined to evaluate the filling capacity of SCC in the porous media. A smaller value of inclination indicates a better filling capacity of the SCC. That is to say, an inclination of zero, corresponding to the fluid with zero yield stress such as water, means perfect filling performance.
(2) The theoretical model, $I=(\tau_{0}/(\rho g))(MK(1-\varphi )/s)$, is proposed for evaluation of filling capacity of SCC in porous media. The filling performance of SCC in porous media is determined by the yield stress of the SCC (*τ*_0_), the size (*s*) and the shape (*M*) of the grains in the porous media, and the grain accumulation parameter (*K*). The inclination is directly proportional to the yield stress of the SCC and is inversely proportional to the grain size. The value of *MK* is defined as blocking effect of grains on SCC flowing in the porous media. It only depends on the shape and accumulation of grains and is not affected by the pore scale. The numerical simulation of SCC flowing in the porous media provides consistent results with the theoretical model.
(3) The theoretical model and numerical simulation suggest that a decrease in yield stress of SCC or an increase in size of grains results in better filling performance of SCC in porous media with the same pore ratio. In addition, SCC exhibits better filling capacity in porous media composed of grains with smooth shapes. These points are beneficial for quality control of RFC. It is expected to use rounded large rocks and SCC with low yield stress to ensure good quality of RFC.

## Figures and Tables

**Table 1:** The parameters of the porous boxes.

Porous Box	Shape of Grains	Size of Grains * (mm)	Length of Box (mm)	Height of Box (mm)	Porosity
C_I	Cylinder	*D* = 20.00	312	168	0.644
C_II		*D* = 15.00	307	171	0.663
C_III		*D* = 12.00	310	170	0.660
D_I	Diamond	*a* = 19.05	312	168	0.644
D_II		*a* = 14.28	307	171	0.663
D_III		*a* = 11.43	310	170	0.660
S_I	Square	*c* = 17.72	312	168	0.644
S_II		*c* = 13.29	307	171	0.663
S_III		*c* = 10.63	310	170	0.660

* Note: *D* denotes the diameter of the cylinder; *a* denotes diagonal length of the diamond; *c* denotes the side length of the square.

**Table 2:** The numerical simulation cases and the rheology parameters of SCM.

Porous Box	Case	Spreading Diameter * (mm)	Flow Stoppage Time * (s)	Yields Stress (Pa)	Consistency Index (Pa·s *^m^*) #
C_I	1	222.8	11	28.4	23.6
	2	250.0	13	16.0	23.6
	3	263.0	14	12.4	23.6
	4	296.2	17	6.9	21.2
	5	308.8	19	5.5	18.8
C_II	6	230.6	12	24.0	23.6
	7	246.0	13	17.4	23.6
	8	268.0	14	11.2	23.6
	9	286.0	16	8.1	21.2
	10	306.6	19	5.8	18.8
C_III	11	261.0	14	12.9	23.6
	12	283.8	16	8.5	23.6
	13	304.0	19	6.0	21.2
	14	319.2	20	4.7	18.8

* Deriving from laboratory experiments of Wang et al. [10]. # *m* denotes the power law index of the H–B model.

**Table 3:** Inclination of the final distribution in the nine porous boxes.

Porous Box	Yield Stress (Pa)					
	16.72	18.84	21.2	23.55	28.26	35.33
C_I	0.172	0.188	0.255	0.294	0.325	0.362
C_II	0.215	0.284	0.312	0.359	0.446	0.551
C_III	0.322	0.394	0.447	0.459	0.559	0.674
D_I	0.306	0.349	0.398	0.448	0.494	0.601
D_II	0.379	0.440	0.462	0.530	0.688	0.761
D_III	0.497	0.553	0.621	0.666	0.772	0.979
S_I	0.226	0.260	0.262	0.330	0.390	0.533
S_II	0.331	0.351	0.406	0.462	0.543	0.670
S_III	0.422	0.454	0.492	0.559	0.689	0.870

**Table 4:** The values of parameter *K* for different porous boxes.

Grain Shape	Porous Box	*M*	*s*	*K*	$M\bar{K}$
Cylinder	C_I	4/π	20.00	111.89	152.91
	C_II		15.00	122.50	
	C_III		12.00	125.89	
Diamond	D_I	2.0	19.05	109.18	219.24
	D_II		14.28	110.39	
	D_III		11.43	109.28	
Square	S_I	1.0	17.72	162.94	170.40
	S_II		13.29	174.56	
	S_III		10.63	173.70	
